# Diffusion-weighted imaging lesions after endovascular treatment of cerebral aneurysms: A network meta-analysis

**DOI:** 10.3389/fsurg.2022.964191

**Published:** 2023-01-16

**Authors:** Lijuan Mo, Jianhe Yue, Wanli Yu, Xi Liu, Changhong Tan, Wuxue Peng, Xueying Ding, Lifen Chen

**Affiliations:** ^1^ Department of Neurology, The Second Affiliated Hospital of Chongqing Medical University, Chongqing, China; ^2^ Department of Neurosurgery, The Second Affiliated Hospital of Chongqing Medical University, Chongqing, China; ^3^ Department of Neurology, Shenzhen University General Hospital, Shenzhen, China

**Keywords:** intracranial aneurysms, intravascular devices, thromboembolism, diffusion-weighted imaging, treatment

## Abstract

**Background:**

Thromboembolism is one of the common complications in endovascular treatments including coiling alone, stent-assisted coiling (SAC), balloon-assisted coiling (BAC), and flow-diverting (FD) stents. Such treatments are widely used in intracranial aneurysms (IAs), which usually present as positive lesions in diffusion-weighted imaging (DWI). Whether these adjunctive techniques increase postprocedural DWI-positive lesions after endovascular treatment remains unclear.

**Methods:**

A thorough electronic search for the literature published in English between January 2000 and October 2022 was conducted on PubMed, Medline, and EMBASE. Eighteen studies (3 cohort studies and 15 case–control studies) involving 1,843 patients with unruptured IAs (UIAs) were included. We performed a frequentist framework network meta-analysis (NMA) to compare the rank risks of cerebral thromboembolism of the above four endovascular treatments. The incoherence test was used to analyze the statistical disagreement between direct and indirect evidence. Funnel plots were used to analyze publication bias.

**Results:**

The incidences of DWI lesions in patients who received FD stents, SAC, BAC, and coiling alone were 66.1% (109/165), 37.6% (299/795), 31.1% (236/759), and 25.6% (236/921). The incidence of DWI lesions in patients who received FD stents was higher than that in patients who received SAC [OR: 2.40; 95% CI (1.15, 5.00), *P *< 0.05], BAC [OR: 2.62; 95% CI (1.19, 5.77), *P *< 0.05], or coiling alone [OR: 2.77; 95% CI (1.26, 6.07), *P *< 0.05]. The incoherence test showed preferable consistency in this NMA. No obvious publication bias was found in the funnel plot.

**Conclusion:**

FD stent placement brings more ischemic lesions identified by DWI than any other procedures for patients with UIA. The characteristics of FD stents may result in a high incidence of DWI lesions.

## Introduction

Endovascular treatments, mainly including coiling alone, stent-assisted coiling (SAC), balloon-assisted coiling (BAC), and flow-diverting (FD) stents, have been increasingly used in treating intracranial aneurysms (IAs) (ruptured or unruptured) with favorable efficacy and minimal invasion. However, complications induced by endovascular treatments, including thromboembolic events, cannot be ignored. Cerebral thromboembolism after endovascular treatments can be either silent (asymptomatic) or symptomatic. Silent thromboembolism is rarely noticed because it has no short-term symptoms. Previous studies have found that silent thromboembolism impairs cognition (1, 2). Furthermore, many microemboli presenting as diffusion-weighted imaging (DWI)-positive spots have been reported to be a surrogate marker for symptomatic thromboembolism (3), suggesting a possible association between DWI-positive lesions and cognitive dysfunction, dementia, or clinical infarction, especially in a long-term course (4, 5).

Meanwhile, with the widespread application of DWI, numerous studies have investigated postprocedural DWI-positive lesions after endovascular treatment for IAs. The incidence of silent thromboembolism has been reported to range from 5.5% to 73% (6, 7), which can be attributed to differences in patient conditions, preventive therapies for periprocedural thromboembolism, and scan settings of MRI systems. Intravascular devices may also affect the incidence of periprocedural thromboembolism, but conflicting findings from earlier research have been observed. According to several studies, BAC was reported to cause more silent ischemia than traditional coiling (8). By contrast, some studies have shown that BAC decreases postprocedural DWI-positive lesion incidence (9). BAC was also found to present lower DWI-positive lesions than SAC (3). Other studies reported no significant relationship between adjunctive techniques and silent ischemia in endovascular treatments (10–13).

FD stents are an assisting device introduced within the last two decades for treating large/giant, wide-necked, sidewall, and fusiform aneurysms (14). Whether FD stent placement increases postprocedural DWI-positive lesions is unclear. Bond et al. first performed a systematic review and meta-analysis and revealed no difference in the incidence of DWI-positive lesions between patients who received coiling alone, BAC, SAC, and FD stents, but they reported that FD stent insertion caused more DWI-positive lesions than the three other devices (15). Notably, the lack of direct comparison and significant heterogeneity may lead to bias in the research of Bond et al. (15). Additional findings with low heterogeneity are needed to clarify whether adjunctive techniques increase postprocedural DWI-positive lesions in endovascular treatments. Therefore, we conducted this network meta-analysis (NMA) to analyze the correlation between different endovascular treatments and the DWI-positive lesion risk in patients with IAs.

## Methods

Our NMA was conducted following standard methods from the Cochrane handbook and the PRISMA NMA checklist.

### Search strategy

A systematic electronic search was performed on PubMed, Medline, and EMBASE for the literature published in English from January 2000 to October 2022. The used search terms were (((((((diffusion) OR (restricted diffusion)) OR (magnetic resonance imaging)) OR (Diffusion Weighted Imaging)) OR ((DWI)) OR (Thromboembolic)) AND ((((((coiling) OR (pipeline)) OR (flow diverter)) OR (stent)) OR (balloon)) OR (endovascular))) OR (((((WEB) OR (Woven EndoBridge)) OR (intrasaccular flow diverters)) OR (flow disruptions)) OR (intrasaccular FD)) OR (Contour))) AND ((((aneurysm) OR (intracranial aneurysm)) OR (cerebral aneurysm)) OR (brain aneurysm)). The reference lists of relevant reviews were also checked for potentially eligible studies.

### Study selection

Published studies were included if they fulfilled the following criteria:

1. randomized controlled trials (RCTs), case–control studies, or cohort designs;
2. comparison of cerebral thromboembolism incidence between different endovascular treatments in patients with IA, including coiling alone, BAC, SAC, and FD stents;
3. articles published in English; and
4. participants received DWI within 2 weeks after the procedure.

The exclusion criteria were as follows:

1. studies with a design other than RCT, case–control, or cohort (e.g., case reports, letters, interventional studies, and reviews);
2. studies that analyzed single endovascular treatment; and
3. research lacking DWI examinations after procedures.

We used a two-step procedure for the selection of eligible studies. In the first step, two investigators independently screened titles and abstracts. In the second step, the two investigators evaluated the full texts of the remaining studies. All disagreements were discussed and resolved by consensus with a third researcher.

### Data extraction

The following information items were extracted from the included studies: name of the first author; publication year; study design; location; the number of participants; the number of aneurysms; time for imaging after procedures; the number of aneurysms treated by coiling alone, BAC, SAC, or FD stents; antiplatelet strategy; results of antiplatelet testing; and the number of positive lesions in DWI of these participants (Supplementary Table S1).

The two investigators independently evaluated the quality of the included studies using the Newcastle–Ottawa Scale (NOS). A score of five or higher was generally regarded as denoting high-quality literature. Any discrepancies were settled by consensus following consultation with a third investigator (Supplementary Table S2).

### Statistical analysis

Stata 14.0 software (StataCorp LLC, College Station, USA) was used for data analysis. Odds ratio (OR) and 95% confidence interval (CI) were employed to estimate the results of NMA. OR values with 95% CI for effect estimates did not include 1 or were deemed statistically significant at *P *< 0.05. A random effects model was used to pool the original data. A frequentist NMA of aggregated data was performed to obtain network estimates for the aforementioned outcomes of interest. The inverse variance was used to combine direct evidence from head-to-head comparisons and indirect comparisons in the frequentist framework.

The network plot provided an intuitive description for the comparison of different trials. Direct comparison within research was shown by a line connecting two nodes. Furthermore, line thickness and node size were positively associated with trial numbers of each intervention and comparison, respectively. The lack of connection lines indicated that no study directly compared the two interventions. Additionally, we used the incoherence test locally to analyze the statistical disagreement between direct and indirect evidence. More specifically, we compared the posterior mean deviance contributions of individual data points with the consistency and inconsistency model and node splitting analysis. *P *> 0.05 or 95% CI of inconsistent factors including the null value indicated no significant inconsistency. We also conducted two subgroup analyses based on the time of MRI scanning (≤24 h) and based on the publication date (using only literature published in the recent 10 years), respectively.

A simple graphical display and numerical summary were used to rank the probable risk hierarchy of the four operations of endovascular treatments using the surface under the cumulative ranking (SUCRA). The value of SUCRA ranged from 0 to 1. When the SUCRA value in a treatment reached 0, the treatment was guaranteed to rank first; otherwise, it ranked last. Meanwhile, a funnel plot was used to explore the potential publication bias.

Quantitative data were presented as mean ± SD (standard deviation), and enumeration data were presented as a number or percentage. Data were tested for normal distribution by the Shapiro–Wilk test in GraphPad Prism 9. Independent-sample *t*-tests were used to compare differences between two groups of data that were normally distributed. Wilcoxon rank sum tests were applied for data that did not conform to a normal distribution. Results were judged to be statistically significant at *P *< 0.05.

## Results

### Study selection

**Figure 1:**
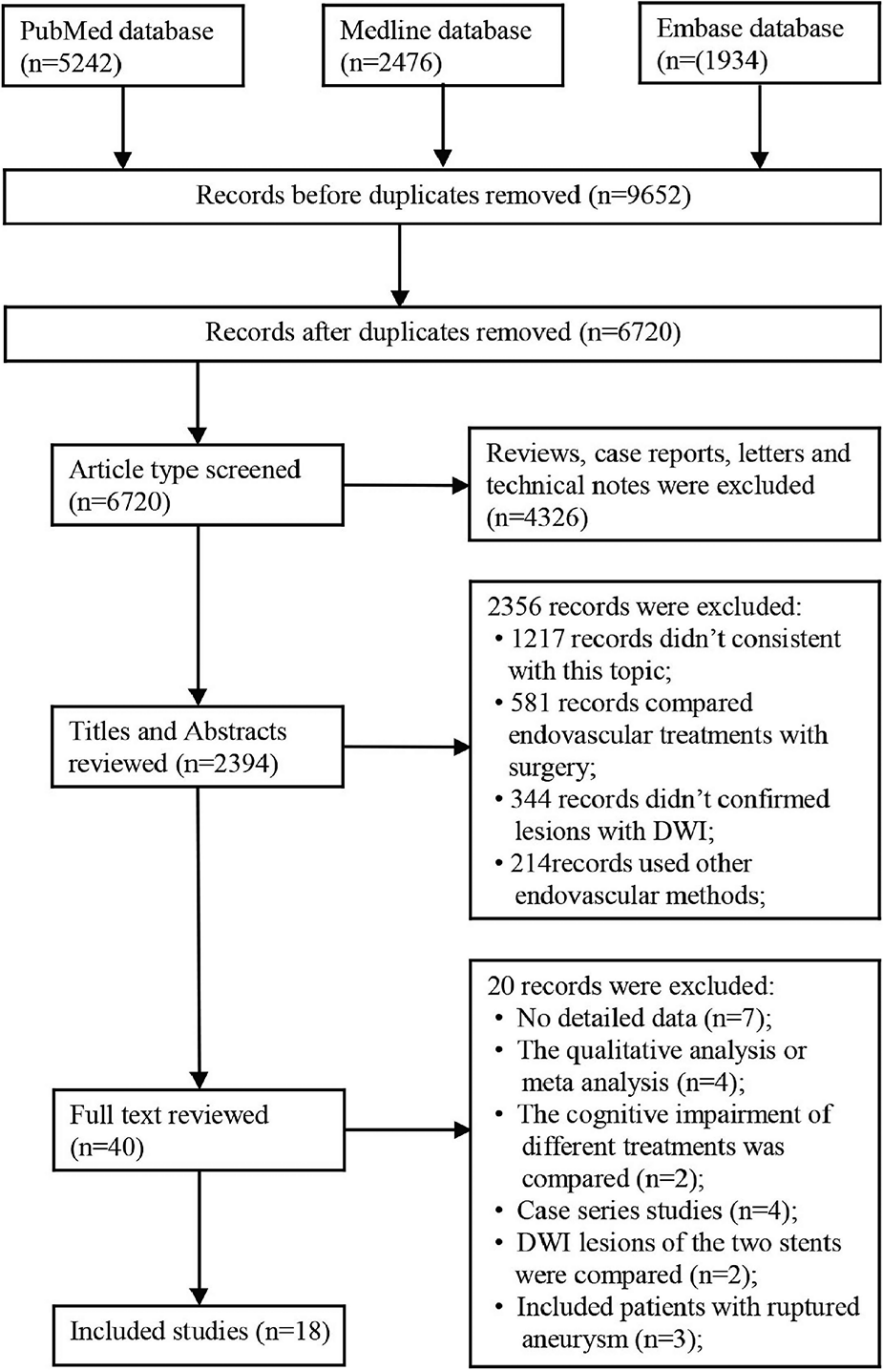
Flowchart of study selection.

A total of 9,652 articles were identified from the three databases (PubMed, Medline, and Embase), among which 2,932 were duplicated. After initial screening, 4,326 articles were excluded due to inappropriate article type. After reviewing the titles and abstracts, 38 eligible studies remained for further screening. Finally, 18 studies containing 2,843 patients with unruptured IAs (UIAs) were included (Figure 1) (2, 9, 12, 16–25).

### Study characteristics

All the included studies (3 cohort studies and 15 case–control studies) were published between 2001 and 2022. Two studies had four eligible arms (18, 22), five studies had three qualified arms (2, 9, 19), and the remaining 11 studies had two eligible arms (12, 16, 17, 20, 21, 23–25). Most participants received DWI within 5 days after operations except Alejandro's (unknown) and Brooks's (≤2 weeks) (2, 12). The sample sizes of the included studies ranged from 14 to 528. All the included studies were of high quality (≥6 stars). Before or after the endovascular treatment procedure, the majority of patients received antiplatelet therapy, which included aspirin alone, clopidogrel alone, or both. In some of the included studies, the response to antiplatelet therapy was routinely assessed using aggregometry to achieve a favorable treatment effect. Subsequent antiplatelet therapy adjustments were performed based on the aggregometry results. All included articles described the administration of systemic heparin during the procedure.

### NMA for risk of DWI lesions

**Figure 2:**
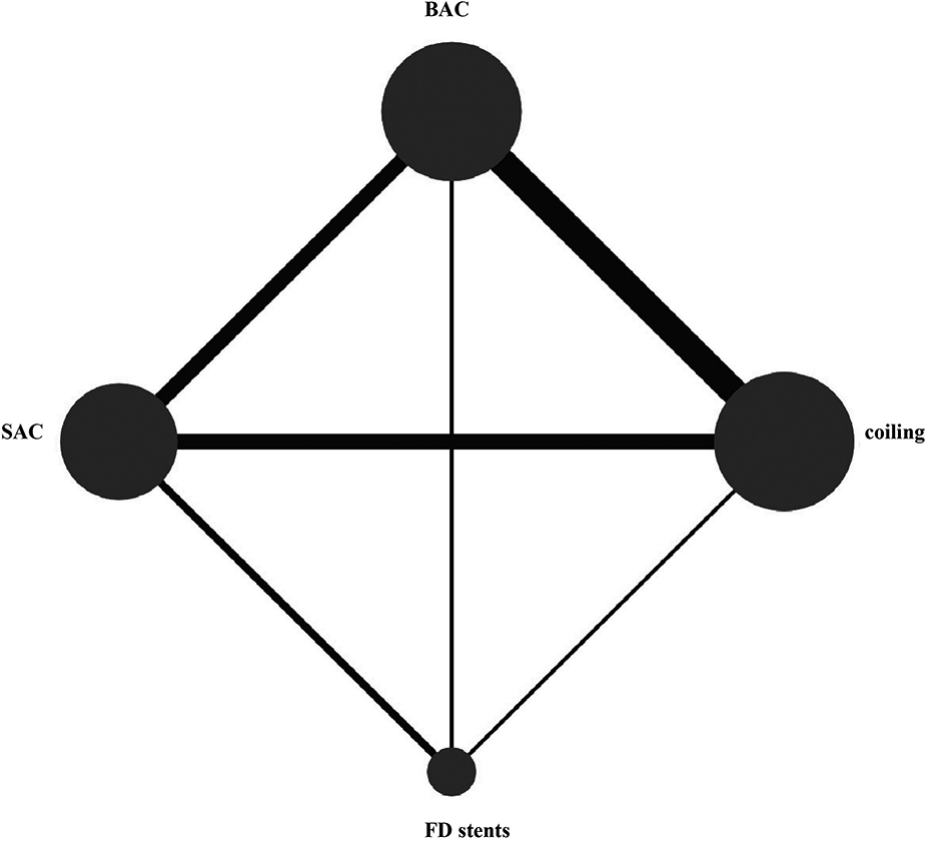
Network diagram of risks in the four endovascular treatments on DWI-positive lesions. BAC, balloon-assistant coiling; SAC, sent-assistant coiling; FD stents, flow-diverting stents.

**Figure 3:**
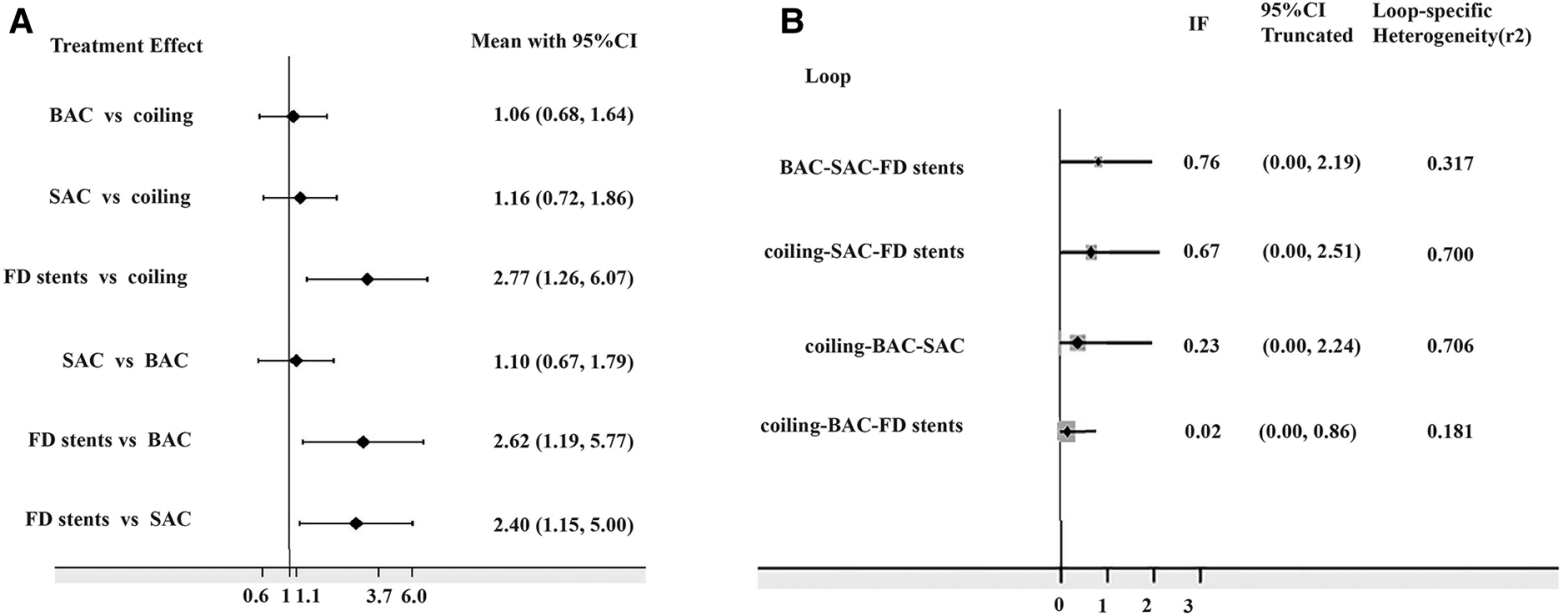
(**A**) Odds risks of different endovascular treatments on DWI-positive lesions; (**B**) inconsistency plot for direct and indirect comparisons. DWI, diffusion-weighted imaging; BAC, balloon-assistant coiling; SAC, stent-assistant coiling; FD stents, flow-diverting stents; CI, confidence interval; IF, inconsistency factor.

The network diagram is presented in Figure 2. The DWI lesion incidence rates in patients who received FD stents, SAC, BAC, and coiling alone were 66.1% (109/165), 37.6% (299/795), 31.1% (236/759), and 25.6% (236/921), respectively. FD stents presented significantly more DWI-positive plots than SAC [OR: 2.40; 95% CI (1.15, 5.00), *P *< 0.05], BAC [OR: 2.62; 95% CI (1.19, 5.77), *P *< 0.05], and coiling alone [OR: 2.77; 95% CI (1.26, 6.07), *P *< 0.05]. No statistical difference was found between coiling alone, SAC, and BAC (*P* > 0.05; Figure 3A and Supplementary Table S3).

**Figure 4:**
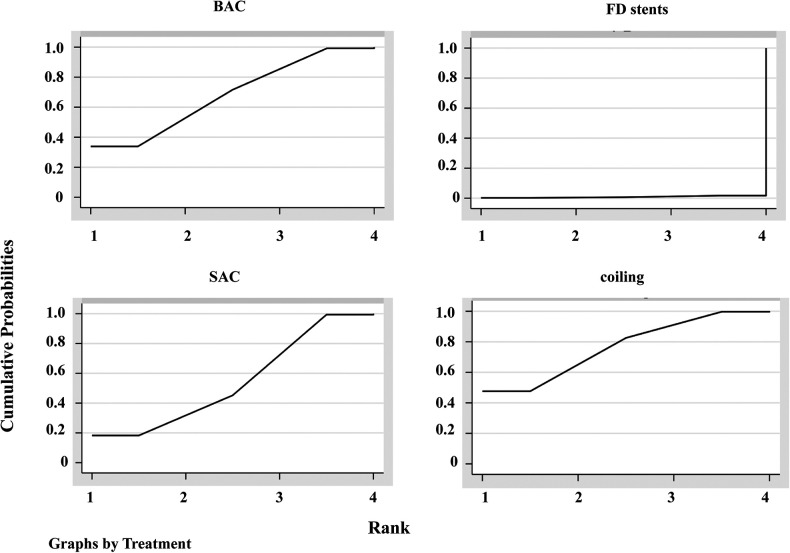
Plot of the surface under the cumulative ranking curves (SUCRA). BAC, balloon-assistant coiling; SAC, stent-assistant coiling; FD stents, flow-diverting stents.

The 95% CI for the inconsistent component included 0 (Figure 3B), indicating that the NMA discovered no evidence of inconsistencies, and all direct and indirect evidence was in agreement. SUCRA reflected the likelihood that DWI lesions will emerge from each treatment method. We concluded that the FD stent mean ranked 4.0, where SUCRA was 0.9%, followed by SAC (mean ranked 2.4 and SUCRA was 54.3%), BAC (mean ranked 2.0 and SUCRA was 68.2%), and coiling alone (mean ranked 1.7 and SUCRA was 76.6%; Figure 4).

The funnel graph showed no obvious asymmetry, indicating small publication bias (Supplementary Figure S1).

### Subgroup analysis

Twelve studies were included for subgroup analysis based on the publication date (published in the recent 10 years) (13, 18, 19, 21–24, 26–29); the DWI lesion incidence rates in patients treated with FD stents, SAC, BAC, and coiling alone were 66.1% (109/165), 39.4% (279/708), 31.1% (191/615), and 22.9% (168/735), respectively. Patients treated with FD stents presented a significantly higher incidence of DWI-positive plots than others (*P *< 0.05). Detailed data are shown in Supplementary Table S4. Nine studies were included for subgroup analysis based on the MRI scanning time (≦24 h) (18, 19, 22–24, 26, 28, 29, 30); the DWI lesion incidence rates of FD stents, SAC, BAC, and coiling alone were 66.1% (109/165), 38.7% (265/684), 42.1% (142/337), and 27.9% (137/490), respectively. The DWI lesion incidence in patients with FD stents was still significantly higher than that in patients treated with SAC and BAC (Supplementary Table S5), and coiling alone presented the lowest DWI lesion incidence among the four endovascular treatments. No significant difference in DWI lesion incidence was found between patients who received FD stents and coiling alone. Detailed data are provided in Supplementary Table S5.

## Discussion

Endovascular treatments have been shown to be extremely effective in treating IAs. However, their potential periprocedural complications, including cerebral thromboembolism, should be considered for reasonable clinical decision-making. Symptomatic cerebral ischemia is usually noticed and treated timely, whereas silent thromboembolism is frequently overlooked (4, 31). In this study, we analyzed 18 studies for the incidence of DWI-positive lesions between different endovascular procedures. DWI lesion incidence rates were 66.1% for FD stents, 37.6% for SAC, 31.1% for BAC, and 25.6% for coiling alone. Among the four endovascular treatments, FD stent placement presented the highest DWI lesion incidence in patients with UIAs. As expected, coiling alone presented the lowest DWI lesion incidence.

DWI lesions or silent microthromboembolic events are common postoperative complications following interventional surgery. A previous study identified that 10%–77% of patients who received endovascular coiling (EC) presented new DWI lesions (32). There are many possible sources of emboli during EC treatment of IAs, including friable plaques and iatrogenic dissection in the parent vessels, air bubbles, electrothrombosis during embolization, hydrophilic coating materials from catheters and wires during catheter insertion of the injection of contrast media or flushing saline, or herniation of the coils into the parent vessel (3, 32–34). Additionally, some researchers demonstrated a higher incidence of DWI lesions in patients with insufficient platelet inhibition (3, 35). Moreover, Yang et al. found that a history of hypertension and hyperlipidemia are risk factors for DWI lesions in patients receiving EC treatment (35). However, whether adjunctive devices increase the frequency of DWI lesions is debatable. According to our findings, coiling alone presented the lowest DWI lesion incidence among the four EC treatments and coiling with adjunctive devices appeared to result in microembolic events, especially FD stents. Fortunately, when appropriate antiplatelet treatment was applied and technique rules were followed, the vast majority of these DWI lesions remained silent and did not appear to hinder the safety of the procedure (36).

FD stents are usually introduced for more complex IAs (particularly thrombosed, giant, wide neck, and fusiform) commonly located in the internal carotid artery or vertebrobasilar arteries. However, their complications should not be underestimated. Brasiliense et al. found that the incidence of DWI-positive lesions after using a pipeline embolization device (PED), an FD stent, was 62.7% (37). Pikis et al. even reported a DWI lesion incidence of up to 90.0% after FD stent treatment (38), whereas the incidence of symptomatic ischemic complications was reported at 0%–13% (39). In our study, we reported that the incidence of DWI lesions reached 66.1% among 165 patients who received FD stents.

Why does the placement of FD stents present such a high DWI lesion incidence rate? First, the metal coverage for FD stents (such as PED, which presents a metal coverage of roughly 30%) is higher than that for traditional stents (the metal coverage of which is typically 5%–16%) (40). In general, lowering device porosity and increasing device pore density result in a greater reduction of intra-aneurysmal flow activity, which theoretically yields increased rates of thrombosis (41). Aneurysms experience a significant change in the ingress/egress flow pattern across the stent struts when stents are deployed over the aneurysm neck. Shear-induced platelet activation may lead to platelet plug initiation and microthrombus formation, both within the aneurysm itself and possibly within the stent-containing parent artery, resulting in distal embolization (37, 42). Therefore, FD stents have a greater possibility to activate platelet and induce microthrombus formation due to their higher metal wire deployment over the aneurysm neck. Using stents with lower metal coverage is reasonable for reducing the DWI lesion incidence when the aneurysm can be successfully occluded.

Notably, timely and proper administration of antiplatelet drugs is necessary for patients receiving stent insertion, especially FD stents, considering that even dual antiplatelet therapy (DAPT) can only partially prevent the mechanical shear gradient platelet activation caused by stents (37). Additionally, the stents are positioned inside the vascular wall permanently, which can also increase platelet reactivity, even after the procedure is completed (43). Therefore, long-term monitoring and managingthe risk of embolism in patients receiving stent insertion are crucial.

Second, FD stent placement frequently requires auxiliary catheters with a large diameter and lengthy procedure time, which are also associated with increased catheter manipulation time and increased risk of catheter- and mechanical manipulation-related thromboembolic events (19, 23, 38).

Third, an ex vivo study reported that all protection devices damage the vessel wall histologically, which commonly results in dislodged debris during the deployment and retrieval of the devices (44). In particular, FD stents have higher hardness and poor adaptability to the twisty artery than traditional stents. Thus, repeated position adjustment may be required during FD stent placement. This additional step may increase the risk of mechanical endothelial damage to the parent artery, which would lead to an increase in thromboembolism and DWI hits (45).

Additionally, traditional methods of coiling (with or without stents and with or without balloon-assisted techniques) aim to exclude the aneurysm from circulation with minimal or even no flow within the aneurysm at the end of the treatment procedure. However, FD stents redirect blood flow, thereby causing stasis of blood flow within the aneurysm and further resulting in thrombosis, inflammatory response, and eventual sealing of the aneurysm neck by endothelialization and neointimal growth with the stent as a scaffold (29). Until the aneurysm is completely occluded by thrombus and aneurysmal neck neointimal formation along the stent, blood still flows in and out of the aneurysm. The increased rate of thrombus egress or escape during the process of transition from implantation until complete healing may contribute to the high DWI lesion incidence of FD stents (29). These characteristics of FD stents may explain the high incidence of DWI lesions associated with FD stents. Numerous factors were reported to influence the incidence of FD stent-associated DWI-positive lesions. For example, aneurysm size, location, and morphology have been reported to affect DWI lesions after PED placement (37, 46). However, in a recent study, Pikis et al. identified that none of these aneurysm characteristics act as statistically significant risk factors for postprocedural silent cerebral ischemia (38). Unfortunately, we did not perform a subgroup analysis for these possible risk factors due to data limitations. But we used limited data to perform a *t*-test and found that there is no significant difference in age, sex, hypertension, smoking, aneurysm size, and location between patients with and without DWI lesions (Supplementary Table S6). More studies are needed to elucidate the risk factors of FD stent-associated DWI lesions.

The debate over the complications of SAC for endovascular treatment of IAs has never stopped since it was introduced. Theoretically, the applications of auxiliary stents and balloons require additional microcatheters and lengthy procedure times. Multiple guiding catheters require stiffer and larger caliber support catheters than single guiding catheters, which increases the possibility of dislodging thrombus and introducing air bubbles or hydrophilic coating materials during the procedure (8). In this NMA, we discovered that patients who received SAC treatment presented a higher incidence of DWI-positive lesions than those who received coiling alone. Consistently, Zhang et al. recently reported that SAC presents a higher incidence of DWI abnormalities than coiling alone (54.4% vs. 45.6%), although no statistically significant difference was found (47). More RCTs are needed to investigate the incidence of DWI lesions in patients receiving SAC treatment. In our included studies, all patients treated with SAC received regular antiplatelet therapy and/or platelet function tests. As a result, we believe that regular antiplatelet therapy may only cause a minor difference in DWI lesion incidence between patients who received SAC and those who received coiling alone. Unfortunately, we failed to perform a subgroup analysis to further confirm the effect of antiplatelet therapy on DWI lesion incidence due to the inconsistency of antiplatelet dosage and duration of antiplatelet therapy in the included literature.

BAC involves the temporary inflation of a compliant balloon in front of the aneurysm neck during coiling (48). The application of BAC can result in better morphology of coils at the aneurysm neck, which minimally interferes with hemodynamics and vortex blood flow, and, consequently, reduces thrombogenesis (49). BAC also facilitates filling with coils and reduces the procedure time, thereby reducing the risk of thrombogenesis (49). Despite these advantages of BAC, it still presents a higher DWI lesion incidence than coiling alone (15). Coiling alone has been reported to exhibit a relatively low incidence of symptomatic ischemic stroke ranging from 2.3% to 10.4% (50, 51), which may be attributed to its relatively simple operation process and auxiliary catheter system. Our NMA also showed that BAC presented an insignificantly higher DWI lesion incidence than coiling alone. More high-quality RCT studies are needed to analyze the DWI lesion incidence between different endovascular treatments.

The included studies used various endovascular treatments and antiplatelet strategies. High-dose and long-term antiplatelet therapy is usually used after FD stent placement (52). By contrast, a common dose of antiplatelet within a short period is used in patients receiving traditional stent than FD stent treatment (53). For BAC, antiplatelet therapy differs between operators (54). Antiplatelet treatment is often considered unnecessary in coiling alone (55). Regular antiplatelet regimens have been proven to decrease the incidence of ischemic events in patients receiving endovascular treatments (56). With antiplatelet therapy, small thromboemboli can be washed out or dissolved and possibly decrease the risk of brain infarction (57). Notably, FD stent placement brings the highest DWI-positive lesion incidence than others, even if it usually requires high-dose antiplatelet therapy. Thus, even high doses of antiplatelet drugs could not completely eliminate the risk of DWI lesions caused by FD stents.

Interestingly, Kim et al. reported that DWI lesions reverted to a normal appearance on MR images by 3 weeks (58), suggesting that DWI lesions may alleviate or even disappear during the disease course. However, we could only perform a subgroup analysis of DWI scanning within ≦24 h after the operation due to data limitations. Our results showed that FD stents exhibited a significantly higher incidence of DWI lesions than SAC and BAC when DWI scanning was performed within ≦24 h after the operation. Additionally, no significant difference in DWI lesion incidence was found between the FD stent group and the coiling alone group; coiling alone still presented the lowest DWI lesion incidence among the four endovascular treatments.

## Limitation

First, most of the included studies were case–control studies. Their retrospective nature inevitably leads to bias. Second, different antiplatelet therapy regimens may also bring bias, especially considering the close association with the risk of ischemic events (51). Third, whether small-sized DWI-positive lesions caused by endovascular treatment for UIAs are associated with clinical symptoms remains controversial, so long-term follow-up is needed. Finally, we only analyzed the relationship between DWI lesions and different procedures in UIAs due to limited data. The relationship between DWI lesions and endovascular treatments on ruptured aneurysms still need to be further explored.

## Conclusion

For patients with UIA, FD stent placement brings more ischemic lesions identified by DWI than other procedures, particularly coiling alone. The characteristics of FD stents may result in a higher DWI lesion incidence, which could not be completely prevented by antiplatelet therapy. In patients with UIA, the risk of ischemic events should be considered when choosing endovascular treatments, whereas FD stents should be used with more caution, under more intense monitoring, and with timely and effective antiplatelet therapy.

## Data Availability

The original contributions presented in the study are included in the article/**Supplementary Material**; further inquiries can be directed to the corresponding author.
