# Supplementary material for: Diffusion-weighted imaging lesions after endovascular treatment of cerebral aneurysms: A network meta-analysis
Source: Front Surg. 2023 Jan 16;9:964191. doi: 10.3389/fsurg.2022.964191 (PMC9885006; doi:10.3389/fsurg.2022.964191)
Supplement: Supplementary file 1 [file Table1.docx]

| **Supplementary table 1. The characteristics of the included studies** | | | | | | | | | | | | | | | |
| --- | --- | --- | --- | --- | --- | --- | --- | --- | --- | --- | --- | --- | --- | --- | --- |
| **No.** | **Study Year** | **Country** | **Study Design** | **Case Type** | **Sample Size** | **Number of**  **aneurysm** | **Time until**  **Imaging (hr)** | **coiling alone, patients** | **BAC,**  **patients** | **SAC,**  **patients** | **FD,**  **patients** | **coiling alone, DWI+** | **BAC,**  **DWI+** | **SAC, DWI+** | **FD,**  **DWI+** |
| 1 | Waldeck 2022 | Germany | case-contral | R | 67 | 67 | 24 | 26 | 41 | - | - | 9 | 8 | - | - |
| 2 | Kim 2021 | Korea | case-contral | R | 528 | 544 | 24 | 267 | 39 | 238 | - | 21 | 14 | 37 | - |
| 3 | Pierot 2020 | France | cohort | P | 335 | 335 | NR | 169 | 166 | - | - | 2 | 6 | - | - |
| 4 | Tokunage 2019 | Japan | case-contral | P | 355 | 376 | 24 | 88 | 115 | 142 | 31 | 49 | 66 | 91 | 26 |
| 5 | Nakae 2018 | Japan | cohort | R | 52 | 57 | 24 | - | - | 27 | 30 | - | - | 19 | 27 |
| 6 | Iosif 2017 | France | cohort | P | 128 | 164 | 12-24 | 24 | 33 | 34 | 63 | 15 | 18 | 17 | 34 |
| 7 | Park 2016 | Korea | case-contral | R | 271 | 271 | 24 | 59 | 16 | 108 | - | 28 | 9 | 51 | - |
| 8 | Safain 2016 | USA | case-contral | P | 119 | 119 | 24 | - | - | 49 | 41 | - | - | 7 | 22 |
| 9 | Seo 2014 | Korea | case-contral | R | 86 | 86 | 24 | 26 | 17 | 43 | - | 15 | 4 | 18 | - |
| 10 | Takigawa 2014 | Japan | case-contral | R | 119 | 119 | 24 | - | 76 | 43 | - |  | 23 | 25 | - |
| 11 | Kim 2014 | Korea | case-contral | R | 58 | 62 | 48 | 34 | - | 24 | - | 14 |  | 14 | - |
| 12 | Nishikawa 2013 | Japan | case-contral | R | 150 | 157 | 48-120 | 42 | 112 | - | - | 15 | 43 | - | - |
| 13 | Alejandro 2011 | American | case-contral | R | 145 | 145 | NR | 66 | 79 | - | - | 15 | 20 | - | - |
| 14 | Altay 2011 | USA | case-contral | R | 184 | 133 | 72 | 41 | 16 | 46 | - | 14 | 3 | 14 | - |
| 15 | Brooks 2008 | British | case-contral | R | 132 | 93 | ≤2 weeks | 24 | 19 | 41 | - | 2 | 2 | 6 | - |
| 16 | Ishibashi 2006 | Japan | case-contral | R | 74 | 45 | ≤48 | 23 | 22 | - | - | 16 | 14 | - | - |
| 17 | Soeda 2003 | Japan | case-contral | R | 26 | 26 | 48-120 | 23 | 3 | - | - | 15 | 3 | - | - |
| 18 | Rordorf 2001 | USA | case-contral | R | 14 | 14 | 48 | 9 | 5 | - | - | 6 | 3 | - | - |

No.=number; hr=hour; BAC= Balloon-assisted coiling; SAC= Stent-assisted coiling; FD=Flow-diverter; DWI=diffusion-weighted imaging; P=prospective; R=retrospective.
