# Supplementary material for: Diffusion-weighted imaging lesions after endovascular treatment of cerebral aneurysms: A network meta-analysis
Source: Front Surg. 2023 Jan 16;9:964191. doi: 10.3389/fsurg.2022.964191 (PMC9885006; doi:10.3389/fsurg.2022.964191)
Supplement: Supplementary file 2 [file Table2.docx]

| **Supplementary table 2. Quality assessment according to the Newcastle-Ottawa Scale** | | | | | | | | | | |
| --- | --- | --- | --- | --- | --- | --- | --- | --- | --- | --- |
| **a. Cohort studies** | | | | | | | | | | |
| **Study**  **(cohort)** | **Representativeness of PPI users** | **Selection of the comparative non-PPI users** | **Ascertainment of the PPI use** | **Demonstration that outcome of interest was not present at start of study** | **Comparability between patients (adjust the most important factor)** | **Comparability between patients (adjust any other factor)** | **Assessment of outcome with independency** | **Adequacy of Follow up length (to assess outcome)** | **Lost to follow up acceptable** | **Sorce** |
| Nakae, 2018 | ⭐ | ⭐ | ⭐ | ⭐ | ⭐ | ⭐ | ⭐ | ⭐ | ⭐ | 9 |
| Iosif, 2017 | ⭐ | ⭐ | ⭐ | ⭐ |  |  | ⭐ | ⭐ | ⭐ | 7 |
| Pierot 2020 | ⭐ | ⭐ | ⭐ | ⭐ |  |  | ⭐ | ⭐ | ⭐ | 7 |

**b. Case-contral studies**

| **Study**  **(case-contral)** | **adequate definition**  **Of dementia** | **representativeness of dementia**  **patient** | **Selection of Controls** | **Definition of Controls** | **Comparability between case and control (adjust the most important factor)** | **Comparability between case and control (adjust any other factor)** | **Ascertainment of exposure** | **Same method of ascertainment for cases and controls** | **Non-Response rate** | **Sorce** |
| --- | --- | --- | --- | --- | --- | --- | --- | --- | --- | --- |
| Park 2016 | ⭐ | ⭐ |  | ⭐ |  |  | ⭐ | ⭐ | ⭐ | 6 |
| Takigawa 2014 | ⭐ | ⭐ |  | ⭐ | ⭐ |  | ⭐ | ⭐ | ⭐ | 7 |
| Kim 2014 | ⭐ | ⭐ |  | ⭐ | ⭐ |  | ⭐ | ⭐ | ⭐ | 7 |
| Alejandro,  2011 | ⭐ | ⭐ |  | ⭐ | ⭐ |  | ⭐ | ⭐ | ⭐ | 7 |
| Nagahata,  2011 | ⭐ | ⭐ |  | ⭐ | ⭐ |  | ⭐ | ⭐ | ⭐ | 7 |
| Altay 2011 | ⭐ | ⭐ |  | ⭐ |  |  | ⭐ | ⭐ | ⭐ | 6 |
| Brooks 2008 | ⭐ | ⭐ |  | ⭐ |  |  | ⭐ | ⭐ | ⭐ | 6 |
| Ishibashi, 2006 | ⭐ | ⭐ |  | ⭐ |  |  | ⭐ | ⭐ | ⭐ | 6 |
| Soeda, 2003 | ⭐ | ⭐ |  | ⭐ | ⭐ |  | ⭐ | ⭐ | ⭐ | 7 |
| Rordorf, 2001 | ⭐ | ⭐ |  | ⭐ |  |  | ⭐ | ⭐ | ⭐ | 6 |
| Tokunage, 2019 | ⭐ | ⭐ | ⭐ | ⭐ |  | ⭐ | ⭐ | ⭐ | ⭐ | 8 |
| Lee 2018 | ⭐ | ⭐ | ⭐ | ⭐ |  |  | ⭐ | ⭐ | ⭐ | 7 |
| Kim 2021 | ⭐ | ⭐ | ⭐ | ⭐ |  |  | ⭐ | ⭐ | ⭐ | 7 |
| Safain 2015 | ⭐ | ⭐ | ⭐ | ⭐ |  | ⭐ | ⭐ | ⭐ | ⭐ | 8 |
| Seo 2014 | ⭐ | ⭐ | ⭐ | ⭐ |  |  | ⭐ | ⭐ | ⭐ | 7 |
| Waldeck 2022 | ⭐ | ⭐ | ⭐ | ⭐ |  | ⭐ | ⭐ | ⭐ | ⭐ | 8 |
