# Supplementary material for: Diffusion-weighted imaging lesions after endovascular treatment of cerebral aneurysms: A network meta-analysis
Source: Front Surg. 2023 Jan 16;9:964191. doi: 10.3389/fsurg.2022.964191 (PMC9885006; doi:10.3389/fsurg.2022.964191)
Supplement: Supplementary file 3 [file Table3.docx]

| **Supplementary table 4. Results of the network meta-analysis** | | | |
| --- | --- | --- | --- |
| **Flow diverter stents** |  |  |  |
| 2.40 (1.15,5.00) * | **Stent-assistant coiling** |  |  |
| 2.62 (1.19,5.77) * | 1.10 (0.67,1.79) | **Balloon-assistant coiling** |  |
| 2.77 (1.26,6.07)* | 1.16 (0.72,1.86) | 1.06 (0.68,1.64) | **Coiling alone** |

* Significant pairwise comparison.
