# Supplementary material for: Diffusion-weighted imaging lesions after endovascular treatment of cerebral aneurysms: A network meta-analysis
Source: Front Surg. 2023 Jan 16;9:964191. doi: 10.3389/fsurg.2022.964191 (PMC9885006; doi:10.3389/fsurg.2022.964191)
Supplement: Supplementary file 4 [file Table4.docx]

| **Supplementary table 4. Results of the network meta-analysis form recent 10 years studies** | | | |
| --- | --- | --- | --- |
| **Flow diverter stents** |  |  |  |
| 2.48 (1.08,5.67)* | **Stent-assistant coiling** |  |  |
| 2.56 (1.03,6.33)* | 1.03 (0.55,1.92) | **Balloon-assistant coiling** |  |
| 2.85 (1.15,7.04)* | 1.15 (0.62,2.12) | 1.11 (0.61,2.02) | **Coiling alone** |

* Significant pairwise comparison.
