# Supplementary material for: Diffusion-weighted imaging lesions after endovascular treatment of cerebral aneurysms: A network meta-analysis
Source: Front Surg. 2023 Jan 16;9:964191. doi: 10.3389/fsurg.2022.964191 (PMC9885006; doi:10.3389/fsurg.2022.964191)
Supplement: Supplementary file 5 [file Table5.docx]

| **Supplementary table 5. Subgroup analysis results of the network meta-analysis (≦24h subgroup)** | | | |
| --- | --- | --- | --- |
| **Flow diverter stents** |  |  |  |
| 2.57 (1.06,6.21) * | **Stent-assistant coiling** |  |  |
| 2.69 (1.01,7.16) * | 1.04 (0.52,2.10) | **Balloon-assistant coiling** |  |
| 2.54 (0.94,6.88) | 0.99 (0.48,2.05) | 0.95 (0.45,1.98) | **Coiling alone** |

* Significant pairwise comparison.
