# Supplementary material for: Diffusion-weighted imaging lesions after endovascular treatment of cerebral aneurysms: A network meta-analysis
Source: Front Surg. 2023 Jan 16;9:964191. doi: 10.3389/fsurg.2022.964191 (PMC9885006; doi:10.3389/fsurg.2022.964191)
Supplement: Supplementary file 6 [file Table6.docx]

| **Supplementary table 6. Characteristics of patients of DWI(+) and DWI (-)** | | | | | | | | | | | | | | | | | | |
| --- | --- | --- | --- | --- | --- | --- | --- | --- | --- | --- | --- | --- | --- | --- | --- | --- | --- | --- |
| **Study Year** | **Total patients** | | **age** | | **Gender（M/F）** | | **hypertension** | | **alcohol consumption** | | **DM** | | **Dyslipidemia** | | **smoking** | | **aneurysm size (mm)** | |
|  | **DWI+** | **DWI-** | **DWI+** | **DWI-** | **DWI+** | **DWI-** | **DWI+** | **DWI-** | **DWI+** | **DWI-** | **DWI+** | **DWI-** | **DWI+** | **DWI-** | **DWI+** | **DWI-** | **DWI+** | **DWI-** |
| Tokunage 2019 | 232 | 144 | 63±11 | 59±13 | 46/186 | 33/111 | 136 | 81 | - | - | 32 | 11 | 84 | 41 | 79 | 39 | 6.8 | 5.6 |
| Park 2016 | 101 | 170 | 60.3 ±9.1 | 55.4 ±10.6 | 27 | 41 | 57 | 68 | - | - | 29 | 22 | 55 | 35 | - | - | 6.08 ±3.38 | 5.57 ±2.73 |
| Kim 2014 | 28 | 30 | - | - | 11/17 | 12/18 | 12 | 11 | - | - | 1 | 3 | 7 | 4 | 8 | 7 | - | - |
| Alejandro 2011 | 20 | 61 | 61.7 ±11 | 55.8 ±11 | 4/16 | 6/55 | 13 | 28 | - | - | 6 | 4 | - | - | 9 | 23 | - | - |
| Kim 2021 | 14 | 25 | 54.6 ±12.2 | 53.2 ±11.2 | 4/10 | 3/22 | 3 | 10 | 1 | 0 | 0 | 1 | 12 | 18 | 2 | 0 | 7.31  ±6.91 | 6.86  ±2.59 |
| Lee 2022 | 17 | 110 | 54.8  ± 9.1 | 52.3 ±11.2 | 12/5 | 60/50 | 5 | 51 | - | - | 1 | 14 | 2 | 12 | 9 | 25 | 14.9  ±5.2 | 11.7  ±6.6 |
| Pierot 2020 | 8 | 335 | 51.7  ±12.3 | 53.3 ±12.2 | 2/6 | 107/228 | - | - | 2 | 70 | 1 | 25 | - | - | 2 | 142 | 6.5  ± 3.2 | 7.1  ± 3.2 |
| Seo 2014 | 37 | 49 | 60.7 ±12.5 | 58.9 ±13.3 | 4/33 | 11/38 | - | - | - | - | - | - | - | - | - | - | - | - |
| P value | - | | 0.32 | | 0.58 | | 0.75 | | - | | 0.43 | | 0.49 | | 0.31 | | 0.65 | |
