# Supplementary figures and images for: Diffusion-weighted imaging lesions after endovascular treatment of cerebral aneurysms: A network meta-analysis
Source: Front Surg. 2023 Jan 16;9:964191. doi: 10.3389/fsurg.2022.964191 (PMC9885006; doi:10.3389/fsurg.2022.964191)

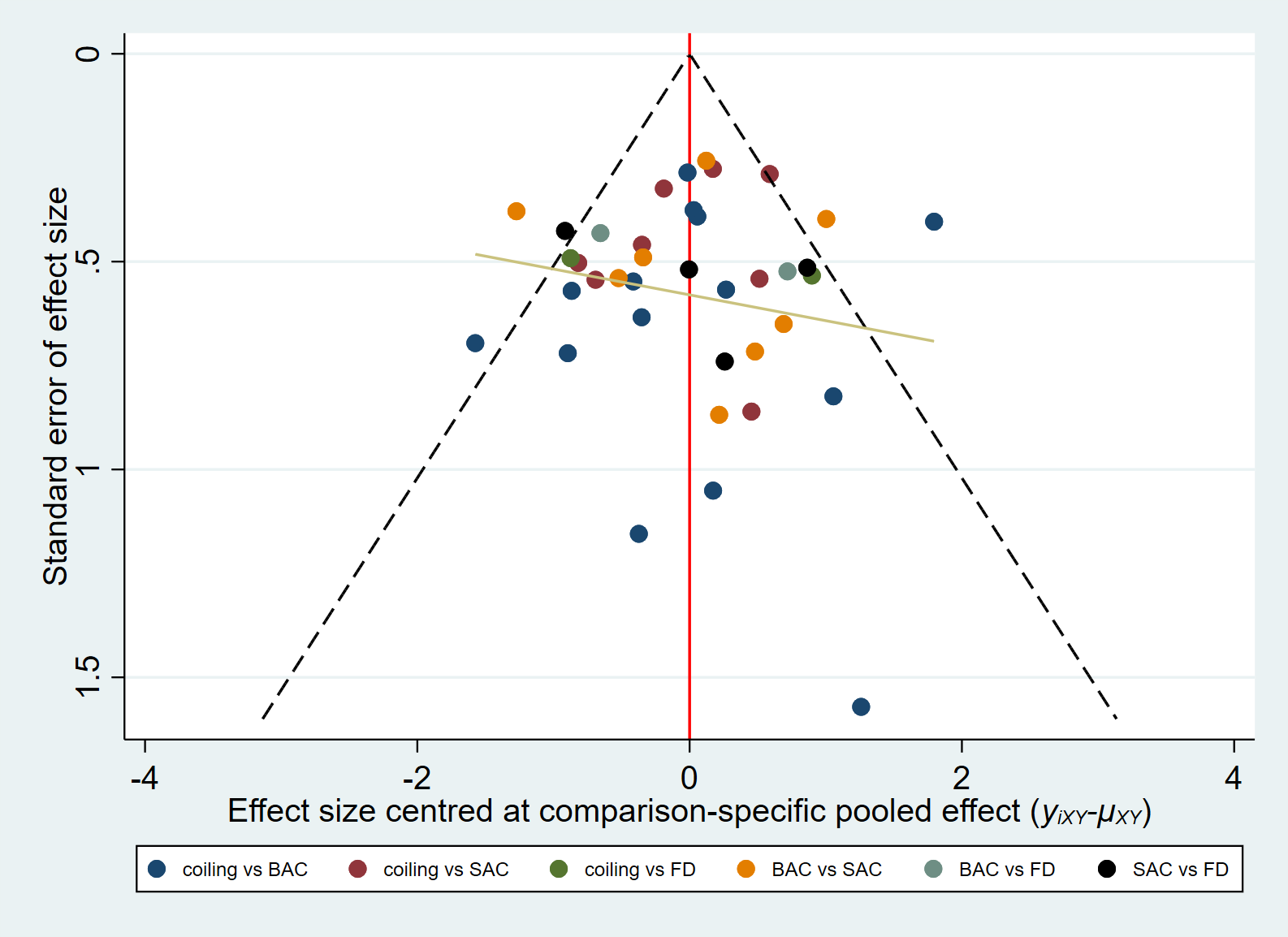

Supplement: Supplementary file 7 [file Image1.tif]
